# A Biomechanical Comparison of Two Techniques of Latarjet Procedure in Cadaveric Shoulders

**DOI:** 10.1155/2020/7496492

**Published:** 2020-01-24

**Authors:** Aditya Prinja, Antony Raymond, Mahesh Pimple

**Affiliations:** Whipps Cross University Hospital, London, UK

## Abstract

Traumatic anterior instability of the shoulder is commonly treated with the Latarjet procedure, which involves transfer of the coracoid process with a conjoint tendon to the anterior aspect of the glenoid. The two most common techniques of the Latarjet are the classical and congruent arc techniques. The aim of this study was to evaluate the difference in force required to dislocate the shoulder after classical and congruent arc Latarjet procedures were performed. Fourteen cadaveric shoulders were dissected and osteotomised to produce a bony Bankart lesion of 25% of the articular surface leading to an “inverted pear-shaped” glenoid. An anteroinferior force was applied whilst the arm was in abduction and external rotation using a pulley system. The force needed to dislocate was noted, and then the shoulders underwent coracoid transfer with the classical and congruent arc techniques. The average force required to dislocate the shoulder after osteotomy was 123.57 N. After classical Latarjet, the average force required was 325.71 N, compared with 327.14 N after the congruent arc technique. This was not statistically significant. In this biomechanical cadaveric study, there is no difference in the force required to dislocate a shoulder after classical and congruent arc techniques of Latarjet, suggesting that both methods are equally effective at preventing anterior dislocation in the position of abduction and external rotation.

## 1. Introduction

Recurrent traumatic anterior instability of shoulder is best managed with operative management [1, 2]. The aim of surgery is to repair the capsule-labral soft tissue structures, and if required, the osseous defects, in order to provide anterior restraint and decrease the capsular volume [3–5]. The Bankart lesion is the most common soft tissue lesion, though variants such as anterior labrum periosteal sleeve avulsion (ALPSA) lesion have been described [6]. Recent arthroscopic techniques have results similar to open procedure with faster rehabilitation and less morbidity [7–10]. However, in the presence of a significant osseous defect, whether humeral (Hill-Sachs lesion) or glenoid (bony Bankart lesion), isolated soft tissue procedures performed either arthroscopic or open have high failure rates [11–14]. The inverse relationship between the size of glenoid defect and the stability of the shoulder has also been established by biomechanical studies [15].

In recent times, addressing the glenoid defect in an attempt to prevent recurrence has gained more attention. The defect can be addressed with a coracoid transfer (the Latarjet procedure), iliac crest bone grafting (the Eden-Hybinette procedure), or other forms of bone graft such as distal tibial allograft [16–19]. The technique of coracoid transfer, first described by Latarjet in 1954, has undergone many modifications. He described a larger (2-3 cm) piece of coracoid transferred over to the glenoid rim lengthwise and fixed with 2 screws to create a robust repair [16]. In 1958, Helfet described attaching the raw cut surface of coracoid process to the glenoid neck through the transversely sectioned subscapularis muscle [17]. He named this procedure after his mentor W. Rowley Bristow, who had taught him this surgery nearly two decades prior. Young et al. published his modifications of the procedure, which included the use of 2 screws instead of 1 to provide stable fixation of the coracoid and a subscapularis-splitting approach [20]. The technique was also modified by De Beer et al. who rotated the coracoid graft about its long axis to line up the concavity of the coracoid with the articular surface of the glenoid (the so-called “congruent arc” technique) [21].

The aim of this study was to compare the biomechanical efficacy of coracoid transfer using the two common techniques, the “classical” and “congruent arc” Laterjet. Both are well described in literature with good clinical results. We hypothesized that the force needed to dislocate the shoulder would be greater in the congruent arc technique than the classical technique because of increased contact surface area as a result of greater linear dimensions [22, 23].

## 2. Materials and Methods

**Figure 1:**
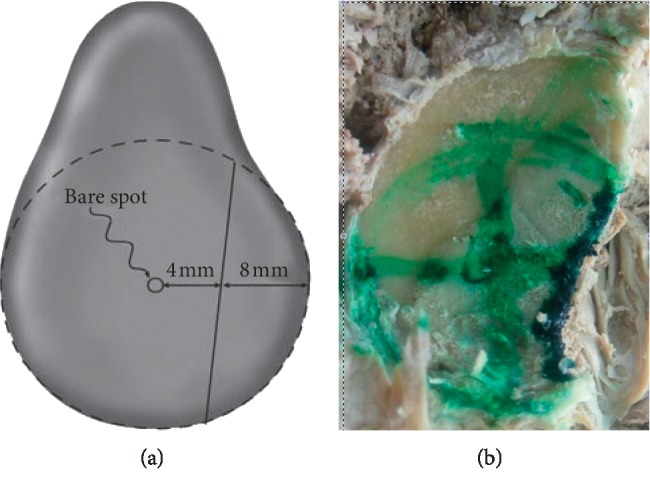
Osteotomy of cadaveric glenoid to recreate “inverted pear-shaped” anterior bony defect. (a) Posterior. (b) Anterior.

**Figure 2:**
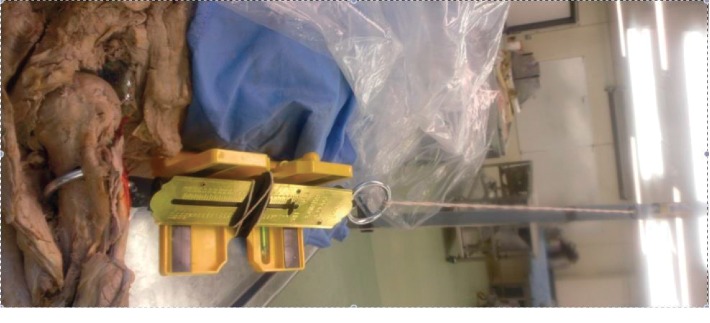
Pulley system attached to the cadaveric upper arm to recreate dislocation force.

We dissected 14 cadaveric shoulders. Deltoid and pectoralis major were detached from their clavicular attachment to improve exposure. Subscapularis and anterior capsule were also detached from their insertion on lesser tuberosity. A bony Bankart lesion was created in the anteroinferior rim of the glenoid. The bony lesions were 25% of longest diameter of the glenoid, to create an “inverted pear-shaped” glenoid (Figure 1) [15, 24]. The osteotomies were made at a 45° inclination to the long axis of glenoid, encompassing ∼8 mm width defect of the inferior glenoid circle. The normal shape of glenoid is of a pear when viewed en face, with lower half significantly wider than the upper half. With a large bony Bankart lesion, the upper half become significantly wider than the lower half, resembling the shape of an inverted pear. A hook was inserted in a drill hole in the lateral humerus just inferior to surgical neck. This was passed over a pulley system incorporating a spring balance. The spring balance had a laser marker and a spirit level system attached to it to recreate the direction of force during each application (Figure 2).

The aim was to generate a force directed anteroinferiorly over the humeral head. The arm was kept in 90 degree of abduction and in maximum external rotation. The pulley system was sequentially loaded until the shoulder dislocated anteriorly. The shoulder was said to be dislocated when it would not relocate after releasing the applied force. The force needed to dislocate was noted.

**Figure 3:**
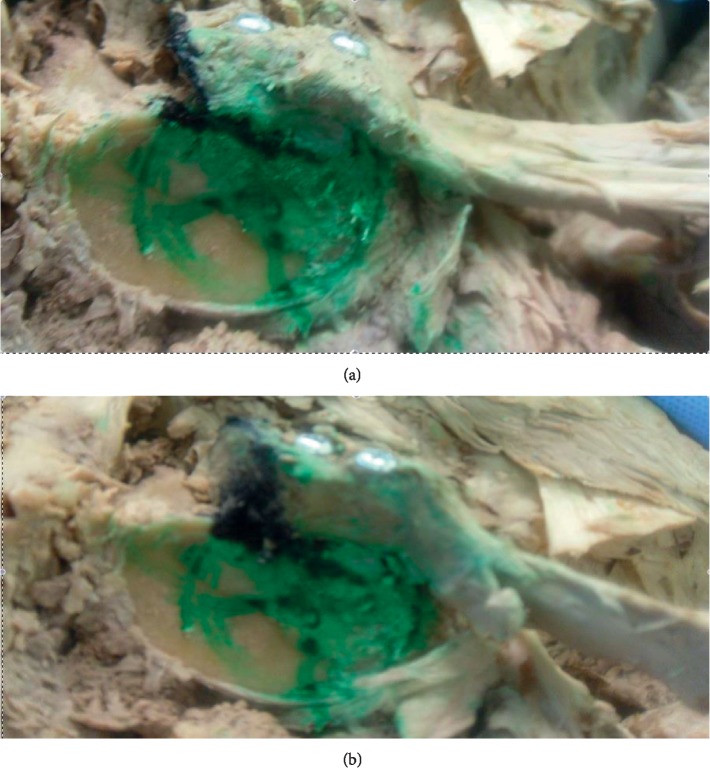
(a) The classical Latarjet. (b) The congruent arc Latarjet.

The coracoid tip was then exposed. The insertion of pectoralis minor was detached. The coracoid was osteotomised at the “knee,” or the junction of horizontal and vertical parts. The graft was then rigidly fixed flush to the anteroinferior glenoid using the classical technique (Figure 3(a)) with two 3.5 mm cortical screws, such that the lateral surface of coracoid became the face of the glenoid [16]. The humeral head was then loaded in a manner similar to that used for the native shoulder before coracoid transfer. The force needed to dislocate the shoulder was noted.

The graft was then removed and reoriented according to the congruent arc Latarjet technique, such that the inferior surface of the coracoid becomes the face of the glenoid [21]. Rigid fixation was confirmed with the application of two 3.5 mm cortical screws. The load was then applied in the similar manner, and the force needed to dislocate shoulder was measured again.

In alternate specimens, the congruent arc Latarjet technique was done first and tested followed by the classical technique. This was done to minimise the effect of any cyclical loading on the biomechanical properties of the construct.

## 3. Results

14 cadaveric specimens were studied. The force required to dislocate uncorrected unstable shoulder was compared with the force required to dislocate the shoulder following “classical” or “congruent arc” Laterjet procedures (Table 1).

A paired *t*-test was used to calculate the difference in mean force needed to dislocate shoulder, before and after the coracoid transfer. The force was calculated with the formula, *F* (in Newton) = load × gravity.

The mean force required to dislocate the shoulder after the classical Latarjet was 325.71 N compared with 123.57 N in the uncorrected shoulder. The standard errors of mean and standard deviation were 6.51 N and 24.37 N, respectively, in uncorrected shoulders. The standard errors of mean and standard deviation were 8.30 N and 31.06 N, respectively, for the shoulders undergoing the classical technique. 95% confidence interval was from −209.00 to −195.28. The two-tailed *P* value was less than 0.0001, thus the difference was statistically significant.

The mean force required to dislocate the shoulder after the congruent arc Laterjet was 327.14 N compared with 123.57 N in the uncorrected shoulder. The standard errors of mean and standard deviation were 6.51 N and 24.37 N, respectively, in uncorrected shoulders, whereas, the standard errors of mean and standard deviation were 7.94 N and 29.72 N, respectively, in the shoulder treated with the congruent arc technique. 95% confidence interval was from −214.57 to −192.57. The two-tailed *P* value was less than 0.0001, thus the difference was statistically significant.

An unpaired *t*-test was performed to compare the force required to dislocate the shoulder treated with the two different techniques. Mean force required to dislocate the shoulder after the classical technique was 325.7 N compared with 327 N after the congruent arc technique. The two-tailed *P* value equals 0.9020 and the 95% confidence interval from −25.05 to 22.19, thus the difference was not statistically significant.

## 4. Discussion

The optimal strategy for surgical stabilization of the unstable glenohumeral joint remains controversial. However, there seems to be an increased awareness that an isolated soft tissue procedure alone is not always appropriate, and addressing osseous defects of a significant size is important to ensure biomechanical stability and good clinical outcomes [13, 25, 26]. The rationale for the Latarjet procedure is that firstly, it provides a “bone block” to fill the void of an anteroinferior glenoid defect and increases the contact surface area of the glenohumeral articulation. Secondly, and crucially, a sling is created by the dynamic support of the repositioned conjoint tendon which supports the humeral head and provides increased stability in abduction and external rotation (the so-called “dynamic sling effect”) [27–30].

The repair of anterior capsulolabral structures has been the standard treatment for traumatic anterior shoulder, since the essential lesion was first described by Perthes, and later by Bankart [31–33]. In Bankart's series of 27 patients, none had bony involvement, leading him to postulate that this was in fact a rare combination [32]. Later, however, Rowe showed in a series of 158 patients, almost three quarters had glenoid rim involvement [33]. The open Bankart procedure, restoring near normal anatomy with low recurrence rates, had been recognized as the gold standard treatment for many years, although the functional outcomes were sometimes reported as suboptimal [34–36]. The arthroscopic Bankart repair was subsequently introduced with the aim of decreasing the morbidity and improving functional outcomes. Despite mixed results initially, advances in arthroscopic techniques have led to widespread uptake of the procedure with good results [8,37–39]. However, several studies have shown increased rates of failures for the arthroscopic procedures, where the significant osseous defects were not addressed [13, 24, 25, 40, 41]. In their study of 194 arthroscopic Bankart repairs, Burkhart et al. [13] reported a 4% recurrence rate in patients without significant bony defects compared with a 67% recurrence rate in those with a significant bony defect (7% vs. 89% in contact athletes, respectively). The study cited a failure to adequately address the glenohumeral osseous defect as the main cause of recurrence.

The glenohumeral defect can be addressed with various methods of coracoid transfer as previously mentioned. In this study, we compared the two most commonly utilised techniques, the “classical” and “congruent arc” Latarjet. In the congruent arc technique, the coracoid graft is rotated about its long axis, and the concavity is lined up with the joint surface [22]. This relatively increases the anteroposterior diameter and hence increases the surface area for anterior translation in comparison with the classic Latarjet technique, where the inferior surface of the coracoid sits on to the anterior inferior rim of glenoid. Furthermore, it has been shown that the coracoid transfer performed using the congruent arc technique restores the glenohumeral loading mechanics to intact condition, while the classical technique restores it within 5% of the intact state [23]. We hypothesized that this relative increase in surface area would make the bony block with the congruent arc technique, a more stable construct in comparison with the classical latarjet technique. This was expected to reflect as an increase in force requirement for dislocation. However, the difference in force needed to dislocate in these two techniques was not statistically significant to establish the superiority of one procedure over the other.

In another cadaveric study by Montgomery et al. significant different loads to failure for the two types of coracoid transfer were demonstrated [42]. They found that the congruent arc technique resulted in a lower mean failure load as compared with the classic technique; however, they were applying a tensile load to the conjoint tendon in a bid to replicate the forces experienced by the graft in the early postoperative period. They remarked that the classic technique created a larger surface area for healing to the native glenoid, whilst the congruent arc produced a greater surface area of the glenoid articular surface. They said, as a result, individual patients' anatomy should be preoperatively considered prior to selecting a technique. Giles et al. in their cadaveric comparison of the two techniques applied medially directed forces across the transferred coracoid to try and replicate the forces across the glenohumeral joint and found that the classic technique failed at a higher load than the congruent arc [43].

Mook et al. demonstrated that assessment of coracoid size preoperatively could predict outcome after Latarjet [44]. They suggested that if predicted glenoid track remained off-track with a classically performed Latarjet, a congruent arc might prove beneficial with its larger surface area. Others have said, however, that larger grafts than necessary will see higher rates of graft osteolysis, as less forces from the humeral head are applied leading to resorption in accordance with Wolff's law [45].

In this study, our results show that there is no statistically significant difference in the force required to produce an anteroinferior dislocation of the shoulder after either classical or congruent arc Latarjet. This suggests that both techniques will provide adequate bony coverage to an anterior glenoid defect and will be effective in preventing recurrent dislocation.

Limitations must be considered when interpreting our results. Firstly, this study was performed on cadavers with a mean age of 84.6 years. This is a procedure most commonly performed on patients who are much younger, and thus the effect of reduced bone mineral density of the grafted coracoid could have affected results. Furthermore, this study did not consider the effect of the conjoint tendon and the dynamic sling effect, and it may be due to the fact that tendon has differing effects according to the position, nor did it consider other soft tissue factors such as capsule-labral repair or the subscapularis split. In this study, we did not aim to address the issue of union of the coracoid, which may also be different, as the techniques differ in the area that is in contact with the glenoid. However, in this study, failure of the construct was solely due to failure of the coracoid transfer itself, and the study has clearly demonstrated that there is no difference in the performance of the transfer using the two techniques.

## 5. Conclusion

To conclude, both the congruent arc and classical technique of coracoid transfer are equally effective in preventing anterior shoulder dislocation in the position of abduction and external rotation in cadaveric specimens.

## Figures and Tables

**Table 1:** Load required to dislocate the shoulder pre- and postcoracoid transfer (with the two techniques).

Cadaver	Load required to dislocate (kg)		
	After osteotomy	Classical Latarjet	Congruent arc Latarjet
1	12	32	33
2	17	39	36
3	9	29	31
4	15	36	37
5	13	32	31
6	11	28	30
7	16	37	38
8	13	33	31
9	10	30	29
10	11	32	32
11	10	30	30
12	12	33	30
13	10	31	34
14	14	34	36

## Data Availability

The data used to support the findings of this study are available from the corresponding author upon request.
